# Navigating coexisting autoimmunity and immunodeficiency in thymoma: a case report of TAMA, Good’s syndrome, and myasthenic crisis

**DOI:** 10.3389/fimmu.2026.1916812

**Published:** 2026-09-09

**Authors:** Youwei Hao, Lizhen Wang, Ke Zheng, Renlong Zhao, Qi Wen, Jianli Wang, Wei Zhang, Juan Wang, Hongxia He, Ruifang Bai, Xueli Chang

**Affiliations:** 1 Department of Neurology, The First Hospital of Shanxi Medical University, Taiyuan, Shanxi, China; 2 The First Clinical Medical College, Shanxi Medical University, Taiyuan, Shanxi, China; 3 Department of Dermatology, The First Hospital of Shanxi Medical University, Taiyuan, Shanxi, China

**Keywords:** Good syndrome, immune deficiency, myasthenia gravis (MG), thymoma, thymoma-associated multiorgan autoimmunity (TAMA)

## Abstract

The coexistence of thymoma-associated multiorgan autoimmunity (TAMA) and Good’s syndrome creates a challenging clinical scenario in which autoimmunity and profound immunodeficiency occur simultaneously. Here, we report a 37-year-old female with invasive WHO Type B3 thymoma and myasthenia gravis (MG) who developed progressive erythroderma-like skin lesions with graft-versus-host disease-like interface dermatitis. Immunological evaluation revealed marked hypogammaglobulinemia, absent circulating B cells, reduced CD4^+^ T cells, and an inverted CD4^+^/CD8^+^ ratio. During a subsequent myasthenic crisis, severe immune dysfunction and active pulmonary infection were already present. Because conventional rapid immunomodulatory options were limited, efgartigimod and eculizumab were administered sequentially. The pulmonary infection continued to progress, and the patient ultimately died. This case highlights the importance of immune and infection risk assessment before potent immunomodulatory therapy.

## Introduction

Thymoma-associated multiorgan autoimmunity (TAMA) is a rare thymoma-associated autoimmune disorder characterized by clinical and histopathological manifestations resembling graft-versus-host disease (GVHD), primarily involving the skin, gastrointestinal tract, and liver. It was first described in 2007 (1–5). Although its exact pathogenesis remains unknown, it is hypothesized that abnormal immune stimulation within thymic tumor cells leads to a loss of immunological tolerance toward self-tissues. Thymoma frequently triggers paraneoplastic autoimmune complications. The most common is myasthenia gravis (MG), diagnosed in 30–50% of patients, followed by Good’s syndrome (5–10%) and pure red cell aplasia (< 5%) (2, 6).

The coexistence of TAMA and Good’s syndrome may result in simultaneous severe autoimmunity and immunodeficiency, posing substantial therapeutic challenges, particularly in the presence of myasthenic crisis and active infection.

Here, we report a patient with recurrent thymoma complicated by TAMA, Good’s syndrome, and myasthenic crisis. Marked immune dysfunction and pulmonary infection were already present before targeted immunotherapy. This case underscores the importance of assessing immune status and infection risk before initiating immunomodulatory treatment in such patients.

## Case report

### Initial presentation and treatment history

A 37-year-old Chinese female presented in July 2018 with progressive limb weakness, ptosis, and dysphagia, and respiratory distress following an infection. Serological testing was positive for anti-acetylcholine receptor antibodies (AChR-Ab) and negative for anti-muscle-specific kinase antibodies (MuSK-Ab). Chest computed tomography (CT) revealed a thymic mass suggestive of thymoma. Based on the characteristic clinical manifestations, AChR-Ab positivity, and chest CT findings, she was diagnosed with thymoma-associated myasthenia gravis (MG). Her condition subsequently progressed to a myasthenic crisis requiring endotracheal intubation and mechanical ventilation. During hospitalization, she received antimicrobial therapy, intravenous immunoglobulin (IVIG), and systemic corticosteroids, after which the myasthenic crisis gradually stabilized. Following discharge, oral prednisone acetate was maintained at 70 mg/day with a planned gradual taper.

On January 23, 2019, the patient underwent extended thymectomy combined with wedge resection of the left upper lobe. Postoperative histopathological examination confirmed WHO type B3 thymoma with invasion of the left upper lobe and phrenic nerve. Her myasthenic symptoms worsened again after surgery. Prednisone acetate was administered at 60 mg/day, and tacrolimus was initiated at 1 mg/day. Thereafter, systemic corticosteroids and tacrolimus were continued as maintenance immunosuppressive therapy.

In September 2019, the patient experienced another exacerbation of MG, characterized by dysarthria, chest tightness, and dyspnea. Imaging evaluation revealed recurrent thymoma; however, the patient and her family did not immediately pursue further oncological treatment. At that time, prednisone acetate had been tapered to 30 mg/day, while tacrolimus had been increased to 3 mg/day. The tumor subsequently progressed, with multifocal metastases involving the left hemidiaphragm and peritoneum. Given the extent of metastatic disease, the patient’s poor general condition, and the absence of an indication for repeat surgical resection, external-beam radiotherapy and systemic chemotherapy were not pursued. Local iodine-125 (^125^I) radioactive seed implantation was therefore selected as a locoregional tumor-control strategy. Four sequential procedures were performed between July 2020 and February 2023, and follow-up imaging showed a slight reduction in the extent of the metastatic lesions. Maintenance therapy with systemic corticosteroids and tacrolimus was continued during this period and gradually tapered.

### Clinical presentation and histopathological findings

**Figure 1:**
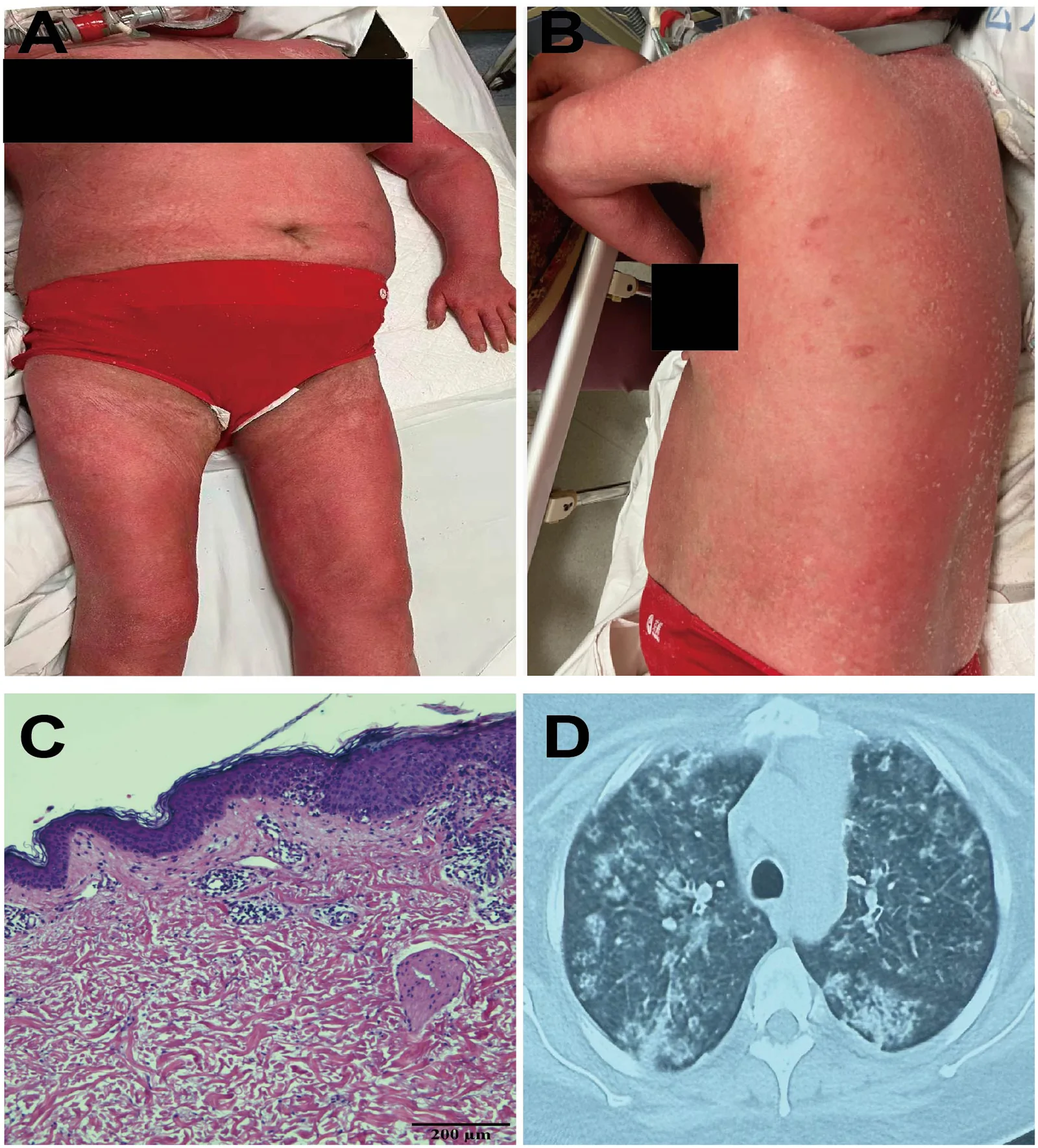
Clinical, pathological, and radiographic features of TAMA: **(A, B)** diffuse, confluent erythema and extensive desquamation, characteristic of exfoliative erythroderma. **(C)** Skin biopsy (H&E) showing hyperparakeratosis, scattered necrotic keratinocytes in the spinous layer, and focal vacuolar interface dermatitis at the basal layer. **(D)** Chest CT demonstrating bilateral diffuse ground-glass opacities and interstitial thickening.

In April 2025, the patient developed a sudden onset generalized skin eruption. Physical examination revealed numerous confluent erythematous maculopapular lesions. During the same period, she experienced recurrent infections accompanied by worsening myasthenic symptoms, requiring intermittent non-invasive ventilatory support. The corticosteroid dose was therefore increased to 30 mg/day, while tacrolimus was maintained at 1 mg/day; however, the clinical response was poor. Between August and September 2025, the cutaneous lesions further progressed to diffuse generalized erythema with extensive desquamation, and erythroderma was diagnosed (**Figures 1A–C**). Prednisone acetate was subsequently increased to 50 mg/day, but the skin manifestations continued to progress. In May 2025, the tacrolimus trough concentration was 5.9 ng/mL.

Notably, the patient reported no gastrointestinal symptoms, denied recent exposure to medications known to induce drug eruptions, and had normal liver enzyme levels. Skin biopsy demonstrated mild hyperkeratosis with parakeratosis, focal necrotic keratinocytes within the spinous layer, and vacuolar degeneration of the basal layer, consistent with histopathological features of graft-versus-host disease (GVHD)-like interface dermatitis. Given her history of thymoma and the absence of prior hematopoietic stem cell transplantation, these clinical and histopathological findings were highly suggestive of thymoma-associated multiorgan autoimmunity (TAMA), prompting further immunological evaluation.

### Immunological findings and diagnosis

**Figure 2:**
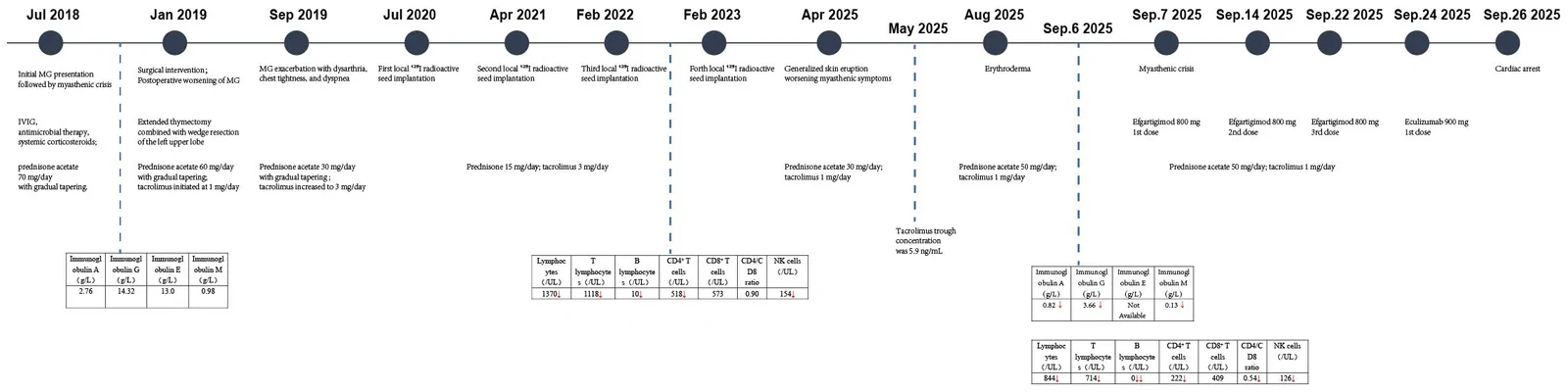
Clinical timeline of disease progression, immunological changes, and treatment.

Further immunological evaluation revealed marked abnormalities in both humoral and cellular immunity. Serum IgG decreased from 14.32 g/L in 2018 to 3.66 g/L on September 6, 2025, accompanied by reduced IgA (0.82 g/L) and IgM (0.13 g/L) levels. Notably, lymphocyte subset analysis performed on February 14, 2022, had already demonstrated substantial immune-cell abnormalities, including a total lymphocyte count of 1,370 cells/μL, markedly reduced circulating B cells (10 cells/μL), a CD4^+^ T-cell count of 518 cells/μL, an NK-cell count of 154 cells/μL, and a CD4^+^/CD8^+^ ratio of 0.90. By September 4, 2025, these abnormalities had further worsened, with a total lymphocyte count of 844 cells/μL, a T-cell count of 714 cells/μL, complete absence of circulating B cells (0 cells/μL), a CD4^+^ T-cell count of 222 cells/μL, an NK-cell count of 126 cells/μL, and an inverted CD4^+^/CD8^+^ ratio of 0.54 (**Figure 2**). These findings, particularly the marked hypogammaglobulinemia, progressive reduction to complete absence of circulating B cells, and associated T-cell abnormalities, were consistent with the characteristic immunological phenotype of Good’s syndrome. Taken together with the recurrent thymoma, characteristic cutaneous manifestations, and GVHD-like interface dermatitis, a final diagnosis of thymoma-associated multiorgan autoimmunity (TAMA) complicated by Good’s syndrome was established.

### Crisis, targeted intervention, and outcome

In early September 2025, the patient developed fever with clinical evidence of pulmonary infection (**Figure 1D**), and sputum culture yielded *Klebsiella pneumoniae*. At that time, her height was 160 cm, body weight was 65 kg, and body mass index (BMI) was 25.39 kg/m². Her neuromuscular symptoms subsequently deteriorated with progressive respiratory muscle weakness consistent with a myasthenic crisis. Endotracheal intubation and invasive mechanical ventilation were recommended because of progressive respiratory failure; however, the patient’s family declined intubation. Respiratory support was therefore provided with non-invasive ventilation. IVIG was considered unsuitable because of the concomitant lower-extremity deep venous thrombosis and associated thrombotic risk, whereas plasma exchange was not pursued after consideration of the patient’s overall critical condition and financial constraints. No immunoglobulin replacement therapy for hypogammaglobulinemia was administered during this hospitalization. Efgartigimod 800 mg was administered on September 7, 14, and 22. Because of the inadequate clinical response and continued deterioration of myasthenic symptoms, the first induction dose of eculizumab (900 mg) was administered on September 24.

Meningococcal vaccination had not been administered before eculizumab because of the urgency of the clinical situation. At that time, the patient was already receiving cefuroxime and cefoperazone/sulbactam sodium for the pre-existing *K. pneumoniae* pulmonary infection; however, no separate antimicrobial prophylaxis specifically for meningococcal infection was instituted. No antifungal or antiviral therapy was administered. Despite antibacterial treatment, non-invasive ventilatory support, and other supportive care, the pulmonary infection continued to progress. The patient developed sudden cardiac arrest and died on September 26, 2025, before the next scheduled dose of eculizumab could be administered.

## Discussion

Thymoma-associated multiorgan autoimmunity (TAMA) is a rare thymoma-associated immune dysregulation syndrome with clinical and histopathological manifestations resembling graft-versus-host disease (GVHD) (5). Thymoma may contribute to the development of TAMA by disrupting the normal thymic microenvironment, impairing antigen presentation and central immune tolerance, and thereby allowing autoreactive T cells to escape into the peripheral circulation (7). In the present case, the patient had a history of WHO type B3 thymoma, followed by postoperative recurrence and metastases involving the left hemidiaphragm and peritoneum. She subsequently developed progressive erythroderma-like skin lesions, and skin biopsy revealed GVHD-like interface dermatitis. In the absence of a history of hematopoietic stem cell transplantation, these clinical and histopathological findings supported the diagnosis of TAMA.

The patient also exhibited immunological abnormalities characteristic of Good’s syndrome. Good’s syndrome is an acquired immunodeficiency associated with thymoma and is primarily characterized by hypogammaglobulinemia and markedly reduced or absent peripheral B cells, often accompanied by reduced CD4^+^ T-cell counts and inversion of the CD4^+^/CD8^+^ ratio (8, 9). In this patient, serum IgG decreased from 14.32 g/L in 2018 to 3.66 g/L on September 6, 2025. On September 4, 2025, circulating B cells were completely absent, accompanied by a reduced CD4^+^ T-cell count and an inverted CD4^+^/CD8^+^ ratio. Notably, marked B-cell reduction, together with decreased CD4^+^ T-cell and NK-cell counts, had already been documented in February 2022, indicating that immune dysfunction predated the clinical deterioration in 2025 and had become more pronounced by 2025. These humoral and cellular immune abnormalities were highly consistent with the characteristic immunological phenotype of Good’s syndrome. However, because serial measurements of immunoglobulin levels and lymphocyte subsets were not available between 2018 and 2025, the precise onset and evolution of the immunodeficiency could not be determined. In addition, the potential effects of long-term corticosteroid and tacrolimus therapy on immune function could not be completely excluded.

The major therapeutic dilemma in this case arose from the coexistence of myasthenic crisis, profound immunodeficiency, and active pulmonary infection. The pulmonary infection preceded the acute neuromuscular deterioration and may have contributed to precipitating the crisis (10). Although IVIG and plasma exchange remain established short-term rescue therapies for myasthenic crisis (11), the available treatment options were substantially limited in this patient. IVIG was considered unsuitable because of the concomitant lower-extremity deep venous thrombosis and associated thrombotic risk, whereas plasma exchange was not pursued after consideration of her overall critical condition and financial constraints. Efgartigimod was therefore used as an alternative therapeutic strategy in this life-threatening and treatment-limited setting, rather than as standard first-line therapy for myasthenic crisis, and eculizumab was subsequently administered because of the inadequate clinical response. Mechanistically, efgartigimod blocks the neonatal Fc receptor (FcRn) and reduces circulating IgG (12), whereas eculizumab inhibits terminal complement activation through blockade of complement component C5 (13). Of particular concern, the patient already had profound hypogammaglobulinemia (IgG 3.66 g/L), absent circulating B cells, and active pulmonary infection before efgartigimod treatment, making the risk–benefit assessment of FcRn blockade particularly challenging. Serial IgG measurements after efgartigimod were unavailable, precluding assessment of any further treatment-associated reduction in IgG. The pulmonary infection continued to progress during the subsequent clinical course. Given the coexistence of multiple infection-related risk factors, including Good’s syndrome, recurrent and metastatic thymoma, and long-term corticosteroid and tacrolimus therapy, the independent contribution of efgartigimod and eculizumab to infection progression and the final outcome cannot be determined. Eculizumab is also associated with an increased risk of invasive meningococcal infection (14), for which meningococcal vaccination is generally recommended before treatment. In the present case, meningococcal vaccination was not administered before eculizumab because of the urgency of the clinical situation.

This case highlights the importance of systematically assessing serum immunoglobulin levels, peripheral B cells, T-cell subsets, and current infection status before initiating potent immunomodulatory therapy in patients with coexisting thymoma-associated autoimmunity and immunodeficiency. Close immunological and infectious monitoring should also be maintained during treatment. Particularly when severe autoimmunity, pre-existing immunodeficiency, and active infection coexist, individualized risk–benefit assessment is essential to balance control of life-threatening autoimmune activity against preservation of adequate anti-infective immunity. Several limitations should be acknowledged. First, the lack of serial immunological monitoring precluded accurate reconstruction of the onset and evolution of immunodeficiency. Second, the administered efgartigimod dose of 800 mg exceeded the recommended weight-based dose of 10 mg/kg for this patient, who weighed 65 kg. Because the specific rationale for this dosing decision could not be fully reconstructed retrospectively, its potential contribution to subsequent immunological deterioration and infection progression remains uncertain. Finally, as a single case report, the relative contributions of baseline immunodeficiency, long-term immunosuppressive therapy, and recent targeted immunotherapy to infection progression and the final outcome cannot be clearly distinguished.

## Conclusion

TAMA complicated by Good’s syndrome represents a complex state of thymoma-associated immune dysregulation characterized by the coexistence of autoimmunity and profound immunodeficiency. This case highlights that, when treating life-threatening autoimmune disease in the setting of severe pre-existing immunodeficiency and active infection, the potential benefits of immunotherapy should be carefully weighed against infectious risks, particularly the risk of progression of pre-existing infection, with comprehensive immune assessment before treatment and close dynamic monitoring throughout therapy.

## Data Availability

The raw data supporting the conclusions of this article will be made available by the authors, without undue reservation.
